# Effective Treatment of Anti‐GAD65 Limbic Encephalitis–Associated Epilepsy Despite Delayed Immunotherapy: A Brief Report

**DOI:** 10.1155/crnm/7926284

**Published:** 2026-07-30

**Authors:** Ghadah Bughaith, Ghadeer Mahdi, Mohammad Ashkanani

**Affiliations:** ^1^ Intern Doctors, Mubarak Hospital, Ministry of Health, Faculty of Medicine, Kuwait University, Kuwait City, Kuwait, kuniv.edu; ^2^ Department of Neurology, Ministry of Health, Al-Amiri Hospital, Kuwait City, Kuwait, amirihospital.com

## Abstract

A 44‐year‐old woman was referred to our epilepsy center for evaluation of refractory seizures persisting for 36 years. Diagnostic workup revealed anti‐glutamic acid decarboxylase 65 limbic encephalitis (anti‐GAD65 LE), supported by markedly elevated serum anti‐GAD65 antibody titers and a compatible clinical presentation. Following initiation of a 6‐month course of intravenous immunoglobulin (IVIG), seizure frequency decreased substantially, from 12 to 16 focal seizures per day to approximately one brief focal seizure per month. This was accompanied by improvements in cognition, psychiatric symptoms, and overall clinical function. This case highlights the potential for meaningful therapeutic response to immunotherapy despite prolonged diagnostic delay.

## 1. Introduction

Anti‐glutamic acid decarboxylase 65 limbic encephalitis (anti‐GAD65 LE) is an autoimmune disorder characterized by antibodies directed against GAD65, an intracellular enzyme expressed in GABAergic interneurons. These antibodies are associated with impaired gamma‐aminobutyric acid (GABA) synthesis, leading to reduced inhibitory neurotransmission and neuronal hyperexcitability [1–3]. Although GAD65 is primarily intracellular, transient surface expression may permit antibody‐mediated effects, and cytotoxic T‐cell mechanisms are also implicated in its pathophysiology [1–3]. Diagnosis is often delayed due to nonspecific or atypical clinical presentations, occasional seronegativity, and overlap with primary epilepsy syndromes [4–6]. Currently, no standardized treatment guidelines exist for patients identified late in the disease course. Here, we describe a patient with long‐standing, treatment‐resistant neurological symptoms who was diagnosed late in the disease course yet achieved marked clinical improvement with immunotherapy, highlighting the potential for meaningful response even in chronic disease.

## 2. Case Summary

Our patient is a 44‐year‐old woman with a 36‐year history of refractory epilepsy and childhood autoimmune disease, including vitiligo, pernicious anemia, and alopecia. She had no recognized epilepsy risk factors, including head trauma, central nervous system infection, or febrile seizures.

At the age of 8 years, she experienced a bilateral tonic–clonic seizure after prolonged sun exposure. Shortly thereafter, she developed three to four daily episodes of behavioral arrest and impaired awareness, occasionally evolving to bilateral tonic–clonic seizures. Within a few months, her seizure burden increased to 20 to 25 episodes per day. Multiple epilepsy monitoring unit and outpatient evaluations demonstrated left mid‐temporal epileptiform discharges, with ictal onset captured on video electroencephalography (EEG). Initial brain magnetic resonance imaging (MRI) was normal, whereas nuclear imaging showed left temporal hypoperfusion. Initial infectious, metabolic, malignant, autoimmune, and connective tissue disease evaluations revealed no direct explanation for her condition, as summarized in Table 1. Lumbar puncture was recommended but declined. She was diagnosed with focal epilepsy with occasional evolution to bilateral tonic–clonic seizures.

**TABLE 1:** Summary of initial serum diagnostic evaluations.

Evaluation category	Tests or assessments included	Result
Infectious	HIV, syphilis serology, hepatitis B and C, tuberculosis testing, Epstein–Barr virus, *Cytomegalovirus*, varicella‐zoster virus	Negative
Metabolic	Thyroid function tests, parathyroid hormone, serum electrolytes, calcium, magnesium, glucose, renal function, liver function, vitamin B12, folate, ammonia, lactate	Vitamin B12 was low in the setting of known pernicious anemia; other tests were within normal limits
Malignancy	Exact testing details are unavailable	Documented as negative in the reviewed records
Autoimmune and connective tissue disease	Antinuclear antibody, extractable nuclear antigen, complement C3 and C4, erythrocyte sedimentation rate, C‐reactive protein, anti‐double‐stranded DNA antibodies	Negative or within normal limits

*Note:* C3, complement component 3; C4, complement component 4; TB, tuberculosis.

Abbreviation: HIV, human immunodeficiency virus.

Between the ages of 8 and 12 years, she received multiple antiseizure medications (ASMs) without meaningful improvement. During this period, she developed progressive neuropsychiatric symptoms, including confusion, aggression, obsessive–compulsive behaviors, and declining academic performance. At the age of 12 years, she underwent left anterior temporal lobectomy and remained seizure‐free for 1 year. Histopathology demonstrated mesial temporal sclerosis. Seizures subsequently recurred and again became refractory. At the age of 17 years, a vagus nerve stimulator (VNS) was implanted without meaningful clinical benefit. Repeat brain MRI around this time demonstrated new contralateral right‐sided mesial temporal sclerosis.

From the ages of 17–42 years, she continued to have refractory seizures with progressive cognitive decline and worsening psychiatric symptoms. She additionally developed new‐onset gait ataxia in her late 30s.

At the age of 42 years, she presented to our center with 12–16 focal seizures per day despite prior epilepsy surgery, VNS, and maximally tolerated ASM therapy. Her medications upon presentation included levetiracetam, lamotrigine, perampanel, clonazepam, valproic acid, and carbamazepine. Given her refractory epilepsy, neuropsychiatric decline, cognitive deterioration, and personal history of autoimmune disease, an autoimmune etiology was reconsidered.

Serum autoimmune encephalitis and connective tissue disease antibody panels revealed markedly elevated anti‐GAD65 antibody titers of 2000 IU/mL, with a reference range of 0–10 IU/mL. Other neuronal surface and paraneoplastic antibodies were negative, as summarized in Table 2. Lumbar puncture was not performed because of patient refusal.

**TABLE 2:** Negative autoimmune encephalitis and paraneoplastic antibody results.

Test category	Antibodies included	Result
Neuronal surface antigen antibodies	NMDAR, LGI1, CASPR2, AMPAR1, AMPAR2, GABABR, DPPX	Negative
Paraneoplastic antibodies	Hu, Ri, Yo, Ma2/Ta, amphiphysin, CRMP5/CV2, SOX1, recoverin, titin	Negative

*Note:* AMPAR, *α*‐amino‐3‐hydroxy‐5‐methyl‐4‐isoxazolepropionic acid receptor; CASPR2, contactin‐associated protein‐like 2; DPPX, dipeptidyl‐peptidase‐like protein 6; GABABR, *γ*‐aminobutyric acid B receptor.

Abbreviations: CRMP5, collapsin response mediator protein 5; LGI1, leucine‐rich glioma‐inactivated 1; NMDAR, N‐methyl‐D‐aspartate receptor.

She was diagnosed with anti‐GAD65 LE and started on intravenous immunoglobulin (IVIG) at 2 g/kg/month, corresponding to 112 g monthly. Corticosteroids were avoided because of concern for worsening psychiatric symptoms. No new ASM was added at IVIG initiation. After the second IVIG infusion, seizure frequency markedly decreased. Given her clinical response, ASMs were gradually reduced during the 6‐month IVIG course from six medications to two. By the end of therapy, her seizure frequency had decreased to approximately one brief focal seizure per month.

IVIG was continued for 6 months and then discontinued according to patient and family preference. Seizure improvement was accompanied by improved psychiatric symptoms and cognition, although gait ataxia persisted. She is currently maintained on valproic acid 250 mg twice daily and lamotrigine 250 mg daily, administered in divided doses. At the most recent follow‐up, 2 years after IVIG treatment, she had sustained seizure control and overall clinical stability. Should relapse occur, reinitiation of immunotherapy will be considered. The major treatment changes and seizure response are summarized in Table 3.

**TABLE 3:** Timeline of treatment changes and seizure response.

Age/period	Treatment/intervention	Seizure response
8–12 years	Multiple ASM trials	Persistent refractory seizures
12 years	Left anterior temporal lobectomy	Seizure‐free for 1 year, then recurrence
17 years	VNS implantation	No sustained benefit
17–42 years	Ongoing ASM adjustments	Persistent refractory seizures with cognitive and psychiatric decline
42 years	IVIG for 6 months	Decreased from 12 to 16 focal seizures/day to approximately one brief focal seizure/month
2‐year follow‐up	Valproic acid and lamotrigine	Sustained seizure control and clinical stability

*Note:* ASM, antiseizure medication; IVIG, intravenous immunoglobulin.

Abbreviation: VNS, vagus nerve stimulator.

## 3. Discussion

The management of anti‐GAD65 LE includes symptomatic treatment with ASMs and psychiatric medications; however, these often fail to achieve adequate disease control [7]. In selected cases, surgical interventions such as VNS and temporal lobectomy may be considered [7, 8]. In our patient, temporal lobectomy resulted in only transient seizure freedom, followed by seizure recurrence and contralateral mesial temporal sclerosis, while VNS provided no meaningful benefit. These findings are consistent with prior reports describing limited long‐term efficacy of surgical interventions in anti‐GAD65–associated epilepsy, particularly when an active immune‐mediated process continues to contribute to epileptogenesis [9, 10].

Immunotherapy is a key treatment strategy and may include corticosteroids, IVIG, and rituximab. In this patient, IVIG was associated with marked and sustained improvement despite reduction of ASM burden from six medications to two. Importantly, no new ASM was added when IVIG was initiated, supporting a likely treatment effect from immunotherapy rather than a change in symptomatic therapy. Her response specifically to IVIG supports evidence that a subset of patients may benefit from humoral‐directed therapy despite GAD65 being an intracellular antigen [11]. Similar outcomes have been reported in delayed diagnoses, suggesting that persistent autoimmune activity may remain therapeutically targetable even decades after onset [11, 12]. Corticosteroids were avoided because of concern for psychiatric worsening, and additional immunotherapy was not pursued given the sustained clinical improvement.

Several factors limit definitive conclusions about this case. The diagnosis was established late in the disease course, as the patient initially presented before autoimmune encephalitis was widely recognized and before anti‐GAD65 testing was available. The absence of cerebrospinal fluid analysis further limits diagnostic certainty, as CSF testing has greater specificity than serum testing alone [1]. Posttreatment antibody titers were not reassessed because of their poor correlation with clinical response [13]. Follow‐up MRI and formal neuropsychological testing were not performed, limiting objective assessment of structural and cognitive outcomes after treatment.

The relationship between the structural and autoimmune findings in this patient can be interpreted in two ways. One possibility is a primary autoimmune process from onset, in which chronic anti‐GAD65–mediated limbic inflammation led to bilateral mesial temporal sclerosis as a consequence of longstanding uncontrolled seizures. Alternatively, this case may represent dual pathology, with initial structural epilepsy followed by a later‐emerging autoimmune process. Regardless of the initial mechanism, autoimmune activity appears to have been a major and treatable driver of disease, as evidenced by the sustained response to immunotherapy. To our knowledge, this represents one of the longest reported delays before initiation of effective immunotherapy in anti‐GAD65 LE, with meaningful improvement despite more than 3 decades of disease.

Two additional observations are notable. First, despite markedly elevated anti‐GAD65 titers and the known association between anti‐GAD65 antibodies and diabetes mellitus, the patient had no clinical history of diabetes mellitus, and hemoglobin A1c testing was within the normal range. Second, her gait ataxia persisted despite treatment, consistent with reports that cerebellar ataxia in anti‐GAD65–associated neurological disease is associated with poorer outcomes [14]. This may reflect an incomplete treatment response or irreversible injury from prolonged disease.

## Author Contributions

Ghadah Bughaith and Ghadeer Mahdi contributed equally to the conception and design of the study, data collection, drafting of the original manuscript, and manuscript revision. Ghadeer Mahdi also served as the corresponding author. Mohammad Ashkanani contributed to the conception and design of the study and supervised the development and revision of the manuscript.

## Funding

No funding was received for this manuscript.

## Ethics Statement

Verbal consent was obtained from the patient for the publication of the details of her medical case.

## Conflicts of Interest

The authors declare no conflicts of interest.

## Data Availability

Some data from this article, including original copies of relevant imaging, lab, and EEG reports, may be shared upon reasonable request to the corresponding author, as long as the data do not contain any identifiable patient information.
